# jEcho: an Evolved weight vector to CHaracterize the protein’s posttranslational modification mOtifs

**DOI:** 10.1007/s12539-015-0260-2

**Published:** 2015-08-06

**Authors:** Miaomiao Zhao, Zhao Zhang, Guoqin Mai, Youxi Luo, Fengfeng Zhou

**Affiliations:** 1 Shenzhen Institutes of Advanced Technology, Key Lab for Health Informatics, Chinese Academy of Sciences, Shenzhen, 518055 China; 2 School of Science, Hubei University of Technology, Wuhan, 430068 China

**Keywords:** jEcho, Evolutionary algorithm, Posttranslational modification (PTM), Motif, Phosphorylation

## Abstract

Protein’s posttranslational modification (PTM) represents a major dynamic regulation of protein functions after the translation of polypeptide chains from mRNA molecule. Compared with the costly and labor-intensive wet laboratory characterization of PTMs, the computer-based detection of PTM residues has been a major complementary technique in recent years. Previous studies demonstrated that the PTM-flanking positions convey different contributions to the computational detection of PTM residue, but did not directly translate this observation into the in silico PTM prediction. We propose a weight vector to represent the variant contributions of the PTM-flanking positions and use an evolutionary algorithm to optimize the vector. Even a simple nearest neighbor algorithm with the incorporated optimal weight vector outperforms the currently available algorithms. The algorithm is implemented as an easy-to-use computer program, jEcho version 1.0. The implementation language, Java, makes jEcho platform-independent and visually interactive. The predicted results may be directly exported as publication-quality images or text files. jEcho may be downloaded from http://www.healthinformaticslab.org/supp/.

**Electronic supplementary material:**

The online version of this article (doi:10.1007/s12539-015-0260-2) contains supplementary material, which is available to authorized users.

## Introduction

Human genome harbors 20,687 protein-coding genes and encodes much larger number of proteins with the help of alternative splicing [1]. After the translation from the mature mRNA, a protein is dynamically modified through various mechanisms and exerts its functions in the dynamically changing modified forms. The posttranslational modification (PTM) of a protein usually introduces a biochemical group to a specific residue, and there are more than 300 types of PTMs [2], e.g., phosphorylation and SUMOylation. Phosphorylation is the major mechanism to deliver the signals between the extra- and intracellular systems [3], and SUMOylation ensures the stability of the modified proteins [4]. Malfunction of PTMs is known to be associated with various human diseases, including cancer and cardiovascular diseases [5]. So a number of PTM types have been extensively studied for their roles in the initiation and development of human diseases.

The PTM residues of proteins may be detected using two major classes of techniques. Both gel- and mass spectrometry-based experimental techniques are widely used to detect the mass change of a peptide after its attachment with the PTM-specific biochemical group, e.g., the 80-Da phosphate group from phosphorylation [6]. Due to the limited availability of catalytic enzymes and low sensitivity, the experimental characterization of PTM residues are still very costly and labor intensive for proteome-wide studies. The alternative strategy is to computationally screen a query protein for residues whose flanking peptides are highly similar to the experimentally verified PTM residues. The current literature supports the assumption that two residues with the same or highly similar flanking peptides tend to have similar probability to be modified by the same PTM type [7]. Various scoring strategies and machine learning algorithms were applied to computationally detect PTM residues from protein sequences [8, 9].

This study proposes a novel position-dependent scoring strategy, the Echo algorithm, to measure the similarity between two peptides. The position-dependent vector of weights for different positions flanking the PTM residues is optimized by an evolutionary algorithm, by simulating the nature’s selection process of random mutation and fitting evaluation. Even the simple nearest neighbor classification strategy based on Echo outperforms similar computer programs for three phosphoserine/threonine kinases, three phosphotyrosine kinases and other three PTM types. A computer program, jEcho, is implemented to facilitate the biologists to easily use these optimized PTM prediction models.

## Materials and Methods

### Data Sources

Experimentally verified phosphorylated residues were collected from the most comprehensive phosphorylation database Phospho.ELM version [10]. The database’s latest version 9.0 was retrieved on July 31, 2012. This study chooses three phosphoserine/threonine kinases (MAPK3, MAPK8 and CDK5) and three phosphotyrosine kinases (EGFR, Met and SYK) as examples to demonstrate how the evolutionary optimization algorithm contributes to PTM residue predictions. In Phospho.ELM version 9.0, there are 91, 33 and 24 phosphorylated residues for MAPK3, MAPK8 and CDK5, respectively. 55, 49 and 26 phosphorylated residues are collected for EGFR, Met and SYK, respectively.

Besides phosphorylation, we also tested our system on three other PTM types, i.e., SUMOylation, Nitrated tyrosine and S nitrosylation. These three PTM data were retrieved from the database dbPTM version 3.0 [11] on November 23, 2012. 1051, 96 and 3289 are collected for the modification types SUMOylation, Nitrated tyrosine and S nitrosylation, respectively.

### PTM Prediction Problem

This study investigates the PTM prediction problem, which is defined as follows. Firstly, for a given PTM type, the modification alphabet is defined to be the amino acid(s) that may be modified by this PTM type. That is to say, {S, T} and {Y} are the modification alphabets for phosphoserine/threonine and phosphotyrosine kinases, respectively. SUMOylation, Nitrated tyrosine and S nitrosylation have the modification alphabets {K}, {Y} and {C}, respectively. The experimentally verified PTM residues of this given PTM type constitutes the positive dataset $P=\{P_{1}, P_{2}, \ldots , P_{G}\}$. A positive data entry is a peptide consisting of *a* upstream, the modified residue and *b* downstream amino acids of the given PTM residue, defined as PSP(*a*, *b*) [7]. The negative dataset $N=\{N_{1}, N_{2}, {\ldots }, N_{H}\}$ are the PSP(*a*, *b*) peptides of all the other residues belonging to the modification alphabet in the proteins with positive residues, as similarly defined in all the other PTM prediction programs [7]. In order to conduct a consistent performance comparison with the program GPS [7], this study uses the same parameters (a = 7 and b = 7) for all the PTM types except SUMOylation. The prediction performance of Echo on SUMOylation is compared with the program SUMOsp [12], so Echo uses the same parameters (a = 5 and b = 5) as SUMOsp.

Echo chooses the simple nearest neighbor algorithm for the PTM prediction problem. The similarity between two PSP(*a*, *b*) peptides *A* and *B* is defined as $Score(A,\;B) = \big \{ \sum \nolimits _{i\in [1,\;a+1+b],\;i\ne a+1} (w_i \times BLOSUM62(A_i, B_i)) \Big \}/(a+b),$ where $w_{i}$ is a predefined weight for the position *i*, and BLOSUM62$(A_{i}, B_{i})$ is the similarity score in the matrix BLOSUM62 [13] between the two amino acids $A_{i}$ and $B_{i}$. For the two datasets *P* and *N*, a query peptide *Q* is defined to be in the same dataset with its nearest neighbor. And the weight vector $W=\{w_{1},w_{2}, {\ldots }, w_{a+1+b}\}$ is optimized by an evolutionary algorithm described in the next section.

### Evaluation Measurements and Evolutionary Algorithm

This study evaluates a PTM prediction algorithm’s performance by its sensitivity (Sn), specificity (Sp), accuracy (Ac) and Matthews correlation coefficient (MCC) [7, 14]. For the positive and negative datasets *P* and *N*, a true positive is a positive data entry predicted to be positive, whereas a positive data entry is a false negative if it is predicted to be negative. A negative data entry is defined to be a true negative and false positive if it is predicted correctly or incorrectly, respectively. The numbers of these classes of data entries are abbreviated as TP, FN, TN and FP, respectively. The algorithm’s prediction performance measurements ${\rm Sn}={\rm TP}/({\rm TP}+{\rm FN}),\; {\rm Sp}={ \rm TN}/({\rm TN}+{\rm FP}),\; {\rm Ac}=({\rm Sn}+{\rm Sp})/2,$ and ${\rm MCC}=({\rm TP} \times {\rm TN-FP} \times {\rm FN})/{\rm sqrt}(({\rm TP}+{\rm FP}) \times ({\rm TP}+{\rm FN}) \times ({\rm TN}+{\rm FP}) \times ({\rm TN}+{\rm FN})),$ where sqrt (*X*) is the squared root of *X*.

An evolutionary algorithm simulates the nature’s random mutation and competitive selection process, and works well on some optimization problems with no clues of optimal patterns [15, 16]. In this work, the weight vector $W=\{w_{1}, w_{2}, \ldots , w_{a+1+b}\}$ is defined to be the molecule that receives the random mutations, and the selection/optimization goal is to maximize the measurement accuracy Ac. Each generation consists of 100 individuals or weight vectors. After the random mutations, 300 pairs of parent individuals are randomly chosen to randomly exchange half positions of their weight vectors. Only the individuals with top 95 Ac values survive or are kept for the next generation. In order to avoid the decrease in Ac in the next generation, the best five individuals are kept intact for the next generation. All the 9 PTM types reach the best Ac values after 1000 generations of optimizations. In case the readers may be interested in the optimized weight vectors, they may be found in the supplementary table S1.

## Results and Discussion

### Comparison of Leave-One-Out Performance

Firstly, we compare the Echo’s prediction accuracy on the three phosphoserine/threonine kinases and three phosphotyrosine kinases with the computer program GPS version 2.1 using the same Jack-Knife validation [14]. The Jack-Knife validation is also called the leave-one-out (LOO) validation, which predicts each data entry’s modification status using all the other data entries as the training dataset [17]. Echo outperforms the GPS 2.1 algorithm in all the four prediction performance measurements in the corresponding cutoff levels for all the six kinases, as shown in Table 1. Even more than 10 % improvements in the overall accuracy Ac values are achieved by Echo for phosphoserine/threonine kinase CDK5 and phosphotyrosine Met, compared with the low cutoff values of the algorithm GPS 2.1. More than 0.20 gain in the Matthews correlation coefficient (MCC) values by Echo for CDK5, EGFR and Met also suggests that Echo performs consistently well on both the positive and negative datasets for these kinases. For example, Echo achieves 100 % accuracy for the positive dataset (Sn) and more than 95 % specificity for the kinases CDK5 and Met, as shown in Table 1. We further evaluate Echo’s performance on identifying phosphorylation residues of six more common kinases, PKA_alpha, MAPK1, Abl, PKG, Aurror_A and ATM. Echo outperforms GPS 2.1 on all the cases with all the threshold values. The maximum improvement 14.04 % in accuracy is achieved by Echo on the low threshold value of kinase Abl.

**Table 1:** Leave-one-out prediction performances of the Echo algorithm compared with the other alternatives

	Cutoff	Sn	Sp	Ac	MCC	Cutoff	Sn	Sp	Ac	MCC
	Echo					GPS 2.1				
MAPK3	2.66	0.6593	0.9598	0.9558	0.3321	High	0.6437	0.9537	0.9492	0.3104
	2.22	0.9451	0.9214	0.9218	0.3485	Medium	0.9310	0.8939	0.8944	0.3027
	2.2	0.9560	0.9198	0.9203	0.3492	Low	0.9425	0.8451	0.8464	0.2503
MAPK8	3	0.4848	0.9781	0.9658	0.4027	High	0.3056	0.9648	0.9497	0.2032
	2.08	0.9697	0.9157	0.9170	0.4490	Medium	0.9444	0.9128	0.9135	0.4150
	1.82	1.0000	0.9071	0.9094	0.4438	Low	0.9444	0.8574	0.8594	0.3264
CDK5	2.3	1.0000	0.9672	0.9678	0.5931	High	0.6316	0.9627	0.9568	0.3684
						Medium	1.0000	0.9206	0.9220	0.4141
						Low	1.0000	0.8651	0.8675	0.3205
EGFR	1.76	0.6909	0.9471	0.9159	0.6191	High	0.6393	0.9410	0.9056	0.5610
	1.53	0.7818	0.9169	0.9004	0.6108	Medium	0.7377	0.8734	0.8574	0.4934
	1.44	0.8727	0.8992	0.8960	0.6374	Low	0.7705	0.8013	0.7977	0.4268
SYK	1.5	0.6735	0.9440	0.9113	0.5970	High	0.5667	0.9386	0.9086	0.4543
	1.3	0.9184	0.9188	0.9187	0.7064	Medium	0.8824	0.8910	0.8900	0.6289
	1.24	0.9184	0.8936	0.8966	0.6559	Low	0.9020	0.8256	0.8349	0.5408
Met	0.94	1.0000	0.9505	0.9606	0.8930	High	0.9615	0.9223	0.9302	0.8126
						Medium	1.0000	0.8252	0.8605	0.6983
						Low	1.0000	0.7961	0.8372	0.6636
PKA_alpha	1.9030	0.7188	0.9804	0.9767	0.4870	High	0.6541	0.9769	0.9714	0.4534
	1.8350	0.8125	0.9603	0.9582	0.4154	Medium	0.8054	0.9415	0.9391	0.3775
	1.8100	0.9375	0.9388	0.9388	0.3957	Low	0.8946	0.9032	0.9030	0.3310
MAPK1	2.3426	0.8333	0.9592	0.9573	0.4264	High	0.7304	0.9533	0.9500	0.3561
	2.0110	0.9250	0.9250	0.9250	0.3608	Medium	0.9130	0.8990	0.8992	0.3090
	2.0096	0.9333	0.9249	0.9250	0.3639	Low	0.9304	0.8470	0.8482	0.2526
Abl	1.6458	0.6250	0.9447	0.9170	0.5234	High	0.4375	0.9170	0.8745	0.3164
	1.6300	0.6458	0.9328	0.9079	0.5058	Medium	0.5208	0.8644	0.8339	0.2915
	1.6000	0.6667	0.9308	0.9079	0.5155	Low	0.5833	0.7854	0.7675	0.2429
PKG	2.2300	0.5909	0.9877	0.9806	0.5141	High	0.5238	0.9866	0.9783	0.4554
	2.2000	0.7273	0.9770	0.9726	0.5026	Medium	0.6190	0.9641	0.9579	0.3673
	2.1000	0.7273	0.9659	0.9617	0.4352	Low	0.6905	0.9360	0.9316	0.3145
Aurror_A	1.3600	0.5517	0.9836	0.9719	0.5028	High	0.3214	0.9746	0.9573	0.2657
	1.3000	0.6897	0.9326	0.9260	0.3641	Medium	0.5714	0.9317	0.9221	0.2956
	1.2130	0.7586	0.9123	0.9082	0.3546	Low	0.6071	0.8888	0.8813	0.2417
ATM	1.7281	0.9123	0.9641	0.9633	0.4885	High	0.8246	0.9628	0.9607	0.4389
	1.7157	1.0000	0.9628	0.9633	0.5238	Medium	0.9649	0.9474	0.9477	0.4413
	1.7000	1.0000	0.9617	0.9623	0.5184	Low	0.9825	0.9443	0.9448	0.4381
	Echo					SUMOsp				
SUMO	3.1838	0.9015	0.9965	0.9923	0.9084	Medium	0.8817	0.9260	0.9243	0.5060
	2.038	0.9284	0.8879	0.8897	0.4731	Low	0.9247	0.8545	0.8572	0.3933
	3.237	0.8925	0.9975	0.9928	0.9138	High	0.8065	0.9670	0.9609	0.6128
	Echo					GPS 3.0				
Nitrated Y	1.9193	0.3125	0.9260	0.8833	0.2114	High	0.2889	0.9002	0.8257	0.1884
	1.7	0.5521	0.8684	0.8464	0.2912	Medium	0.4053	0.8502	0.7960	0.2171
						Low	0.5009	0.8018	0.7651	0.2335
S nitro	2.65	0.3202	0.9921	0.91289	0.48727	High	0.2520	0.9117	0.8040	0.1897
	2	0.48276	0.94273	0.8885	0.44309	Medium	0.3532	0.8672	0.7833	0.2175
	1.69	0.54187	0.83608	0.80139	0.30003	Low	0.5357	0.8014	0.7580	0.2864

The cutoff values of Echo are tuned to match the similar specificity levels of the alternative algorithms, and the four performance measurements are calculated for the Echo algorithm. The performances of the alternative algorithms are collected from the respective publications. Rows of “SUMO” are for SUMOylation, rows of “Nitrated Y” are for Nitrated tyrosine, and rows of “S nitro” are for S nitrosylation

Echo also outperforms the alternative algorithms in any performance measurements for the other three PTM types, i.e., SUMOylation, Nitrated tyrosine and S nitrosylation, as shown in Table 1. A significant improvement has been achieved for S nitrosylation residue predictions. 10.89 % improved Ac and 0.2976 improved MCC for the high cutoff level of S nitrosylation suggest that Echo performs more consistently in both Sn and Sp. Echo improves the overall accuracy Ac by more than 5 % for both SUMOylation and Nitrated tyrosine, and even improves the MCC by 0.4024 for the high cutoff level of SUMOylation. The performance of ${ \rm Sn}=90.15$ % and ${\rm Sp}=99.65$ % for SUMOylation suggests that the annotations of Echo may be reasonably applied to the large-scale characterization of cellular SUMOylation dynamics.

### Fourfold Cross-Validation Performance of jEcho

Reasonable detection performance is also achieved by Echo on all the 15 PTM types using the fourfold cross-validation, as shown in Table 2. As expected, the data of fourfold cross-validation of Echo is slightly smaller than the leave-one-out validation in the above section. But most PTM types receive over 90 % in accuracy by Echo. Echo performs best on the detection of SUMOylation residues, with 99.06 % in the overall accuracy and 0.8857 in MCC, which is even better than the leave-one-out validation of both Echo and GPS on SUMOylation.

**Table 2:** Fourfold cross-validation performance is calculated for all the 15 PTM types

Cutoff	Sn	Sp	Ac	MCC	Cutoff	Sn	Sp	Ac	MCC
MAPK3					MAPK8				
2.3600	0.7473	0.9386	0.8429	0.3083	2.7600	0.4848	0.9711	0.7280	0.3626
2.1000	0.9341	0.9160	0.9251	0.3335	2.0800	0.9697	0.9188	0.9443	0.4564
1.8800	0.9560	0.8988	0.9274	0.3115	1.8200	1.0000	0.9094	0.9547	0.4488
CDK5					EGFR				
2.2900	0.8750	0.9719	0.9235	0.5568	1.5400	0.5636	0.9295	0.7466	0.4785
1.7400	0.9583	0.9236	0.9409	0.4085	1.4400	0.6909	0.9068	0.7989	0.5253
1.4400	1.0000	0.8853	0.9427	0.3525	1.3200	0.7818	0.8564	0.8191	0.5027
SYK					Met				
1.3300	0.6327	0.9412	0.7869	0.5594	2.2800	0.6923	1.0000	0.8462	0.8009
1.2100	0.8163	0.9047	0.8605	0.6085	0.9400	0.7692	0.9505	0.8599	0.7304
1.0900	0.8571	0.8487	0.8529	0.5412	0.6800	0.8462	0.7129	0.7795	0.4601
SUMO					S nitro				
3.0129	0.8597	0.9939	0.9879	0.8573	2.4900	0.4019	0.8462	0.6241	0.2358
2.0380	0.9164	0.8977	0.8986	0.4857	2.1100	0.6072	0.5970	0.6021	0.1554
3.1543	0.8567	0.9968	0.9906	0.8857	1.7500	0.8614	0.2119	0.5366	0.0692
PKA_alpha					MAPK1				
2.4600	0.5000	0.9875	0.7437	0.4169	2.1200	0.7750	0.9409	0.8579	0.3374
1.7900	0.7500	0.9473	0.8486	0.3395	1.9000	0.9167	0.9218	0.9192	0.3505
1.7500	0.9063	0.9419	0.9241	0.3918	1.7900	0.9333	0.9133	0.9233	0.3397
Abl					PKG				
1.5750	0.5833	0.9269	0.7551	0.4460	2.0800	0.6591	0.9704	0.8147	0.4204
1.3900	0.6042	0.8715	0.7379	0.3565	1.5800	0.7955	0.9014	0.8484	0.2930
1.0300	0.7083	0.7233	0.7158	0.2616	1.1300	0.8409	0.7472	0.7941	0.1764
Aurror_A					ATM				
1.2500	0.4483	0.9345	0.6914	0.2350	1.8800	0.4737	0.9802	0.7269	0.3397
1.1800	0.6552	0.9191	0.7871	0.3162	1.2200	0.9825	0.8974	0.9399	0.3305
1.0500	0.6897	0.8767	0.7832	0.2664	1.0500	1.0000	0.8461	0.9231	0.2728
Nitrated Y									
2.8214	0.1563	0.9938	0.9355	0.2981					
1.7246	0.3438	0.9104	0.8710	0.2090					
1.3160	0.4063	0.7189	0.6971	0.0701					

**Fig. 1:**
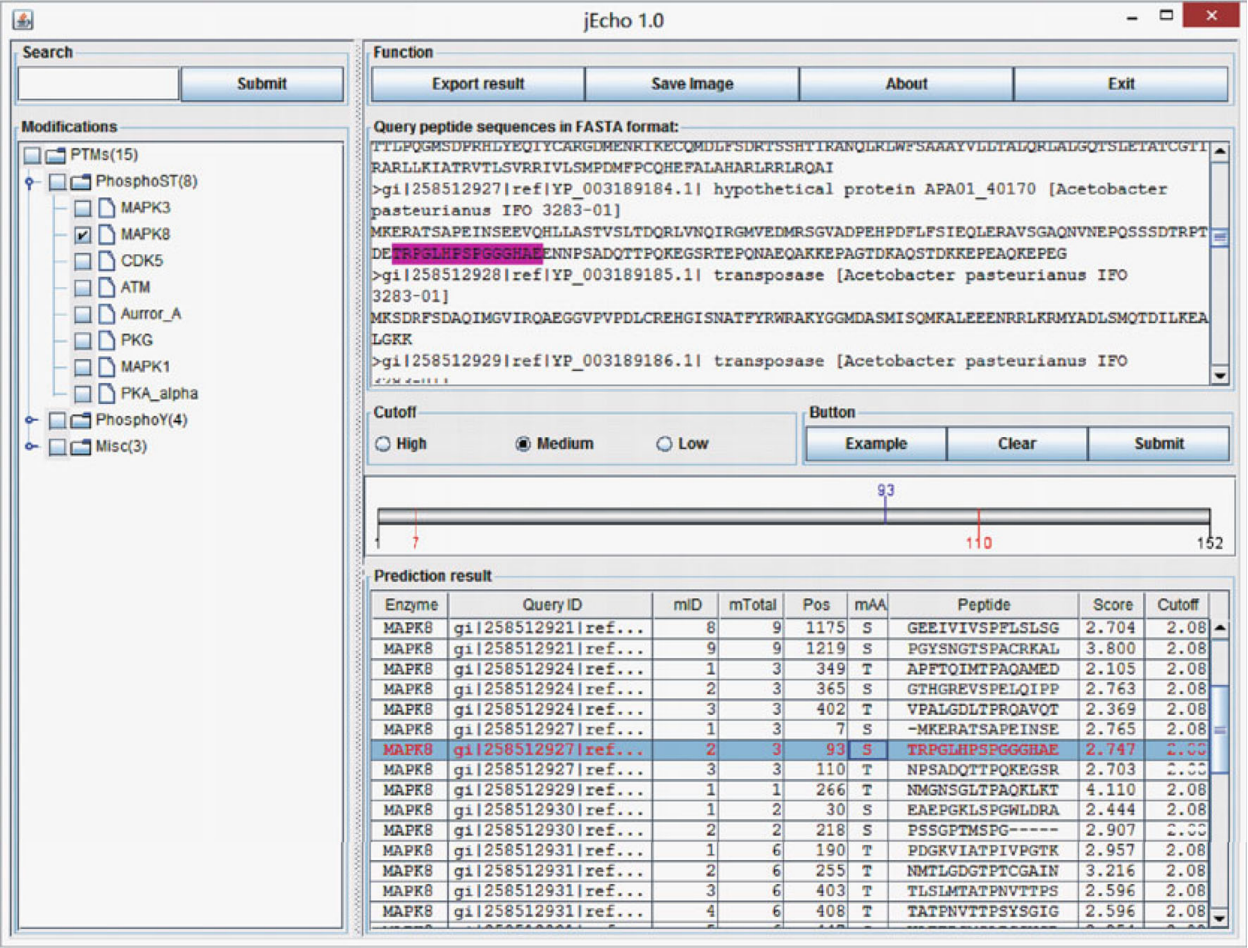
User interface of jEcho version 1.0. The *left tree box* gives the hierarchical list of PTM types. The *top right box* waits for the input of query sequences in FASTA format. The parameters may be tuned in the *right middle box*. The *result box* is in the *bottom right* table. The illustrated data are the predicted from the example proteins by clicking the button “Example”

### Prediction and Visualization of PTM Residues

The Echo algorithm is implemented as an easy-to-use PTM prediction software, jEcho v1, using the Java programming language, as shown in Fig. 1 and Supplementary Figure S1. Firstly, jEcho may be used in any operating systems with a Java running environment. And jEcho is packaged as an JAR file, which contains all the required external libraries. A user may run jEcho directly after downloading it. Secondly, jEcho has an all-in-one user interface (UI), so that a user may get any information from the UI, as the standard of a PTM prediction server/program [8]. Thirdly, after a user generates the PTM predictions for a specific catalytic enzyme, the distribution of all the predicted PTM residues may be visualized in the current protein by clicking the prediction in the right bottom result area, as in Supplementary Figure S1 (d) and (e). Lastly, the predicted results may be exported as a text file or an image file, by clicking a button in Fig. 1 right top area.

## Electronic supplementary material

Below is the link to the electronic supplementary material.

Supplementary material 1 (PDF 655 kb)
