# Supplementary material for: jEcho: an Evolved weight vector to CHaracterize the protein’s posttranslational modification mOtifs
Source: Interdiscip Sci. 2015 Aug 6;7(2):194–9. doi: 10.1007/s12539-015-0260-2 (PMC4551539; doi:10.1007/s12539-015-0260-2)
Supplement: Supplementary file 1 — Supplementary material 1 (PDF 655 kb) [file 12539_2015_260_MOESM1_ESM.pdf]

jEcho: an Evolved weight vector to CHaracterize the protein's post-translational modification mOtifs

Miaomiao Zhao[1,*], Zhao Zhang[1,*],Guoqin Mai[1], Youxi Luo[1,2], Fengfeng Zhou[1,#].

[1] Shenzhen Institutes of Advanced Technology, and Key Lab for Health Informatics, Chinese Academy of Sciences, Shenzhen, Guangdong, P.R. China, 518055.

[2] School of Science, Hubei University of Technology, Wuhan, 430068.

[*] These authors contribute equally to this work.

[#] Corresponding author: Fengfeng Zhou, phone: +86-755-86392200; fax: +86-755-86392299; e-mail: FengfengZhou@gmail.com, or ff.zhou@siat.ac.cn. Web site: http://www.healthinformaticslab.org/ffzhou/ .

Supplementary Table S1

The optimized weight vectors used in the program jEcho version 1.0.

Category	PTM	Optimized weight vector
PhosphoST	MAPK3	0.450556, 0.056809, 0.029316, 0.232540, 0.237927, 0.574702, 0.104020, 0.00, 0.875317, 0.113308, 0.114724, 0.403124, 0.141559, 0.177063, 0.308348
	MAPK8	0.479205, 0.100383, 0.379073, 0.029030, 0.128421, 0.058653, 0.113985, 0.00, 0.812237, 0.559825, 0.171227, 0.118769, 0.045587, 0.046538, 0.016736
	CDK5	0.027765, 0.856953, 0.135015, 0.619904, 0.292570, 0.116146, 0.049196, 0.0, 0.946398, 0.048994, 0.032542, 0.893598, 0.233115, 0.434184, 0.020201
	PKA_alpha	0.143016,0.041709,0.074195,0.032190,0.434228,0.541888,0.094520,0.000, 0.134524,0.252768,0.002140,0.095742,0.068922,0.027074,0.006881
	MAPK1	0.297371,0.340018,0.125696,0.237952,0.339784,0.216438,0.163280,0, 0.826476,0.159929,0.074486,0.464732,0.208783,0.446258,0.220159
	PKG	0.814342,0.374948,0.049688,0.046986,0.839916,0.555832,0.245153,0, 0.027420,0.114112,0.107563,0.134397,0.055669,0.062553,0.396035
	Aurror_A	0.371918,0.449953,0.876654,0.337775,0.122510,0.542665,0.921812,0.000, 0.591022,0.903234,0.160365,0.202245,0.758482,0.461625,0.240552
	ATM	0.072056,0.062092,0.046059,0.040450,0.055920,0.030036,0.113512,0, 0.235401,0.030044,0.071767,0.047614,0.105293,0.047997,0.062171
PhosphoY	EGFR	0.010200, 0.305737, 0.731499, 0.634122, 0.321978, 0.959081, 0.565565, 0.00, 0.215641, 0.284387, 0.032544, 0.292086, 0.065496, 0.306817, 0.908672
	SYK	0.958202, 0.329346, 0.929670, 0.946850, 0.679151, 0.040191, 0.163201, 0.00, 0.004147, 0.488092, 0.005949, 0.945958, 0.802146, 0.549347, 0.674299
	Met	1.000000, 0.514861, 1.000000, 1.000000, 0.925675, 1.000000, 0.842642, 0.0, ,0.151195, 0.023802, 0.218899, 0.136854, 0.068564, 0.740515, 0.412213
	Abl	0.044744,0.050416,0.024242,0.181738,0.290950,0.241188,0.625328,0.000, 0.517711,0.531499,0.756024,0.433096,0.012740,0.652793,0.497044
	SUMOylation	0.485850, 0.318964, 0.419421, 0.463420, 0.450487, 0, 0.344639, 0.722873, 0.968511, 0.990939, 0.257791
	Nitrated tyrosine	0.036558, 0.758844, 0.938228, 0.066790, 0.196185, 0.156633, 0.026869, 0.000, 0.532746, 0.207878, 0.436206, 0.502079, 0.216688, 0.067212, 0.549788
	S nitrosylation	0.119848, 0.749064, 0.547875, 0.116078, 0.221132, 0.218336, 0.974647, 0.000, 1.000000, 0.569575, 0.126067, 0.179458, 0.385199, 0.059752, 0.689456

Supplementary Figure S1

User interfaces of jEcho version 1.0. (a) Initial window. (b) Example result window. (c) Sort window. (d) Residue locating window. (e) PTM residue plotting window. (f) PTM type searching window.

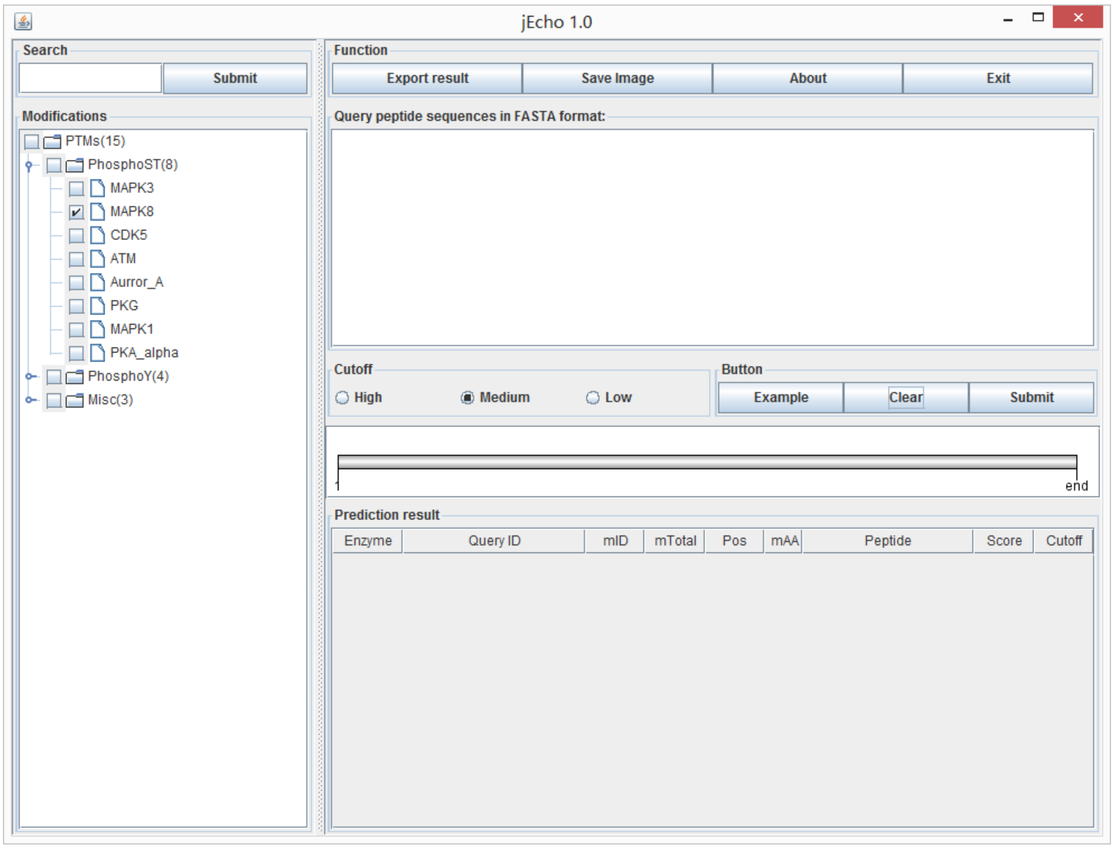

(a)

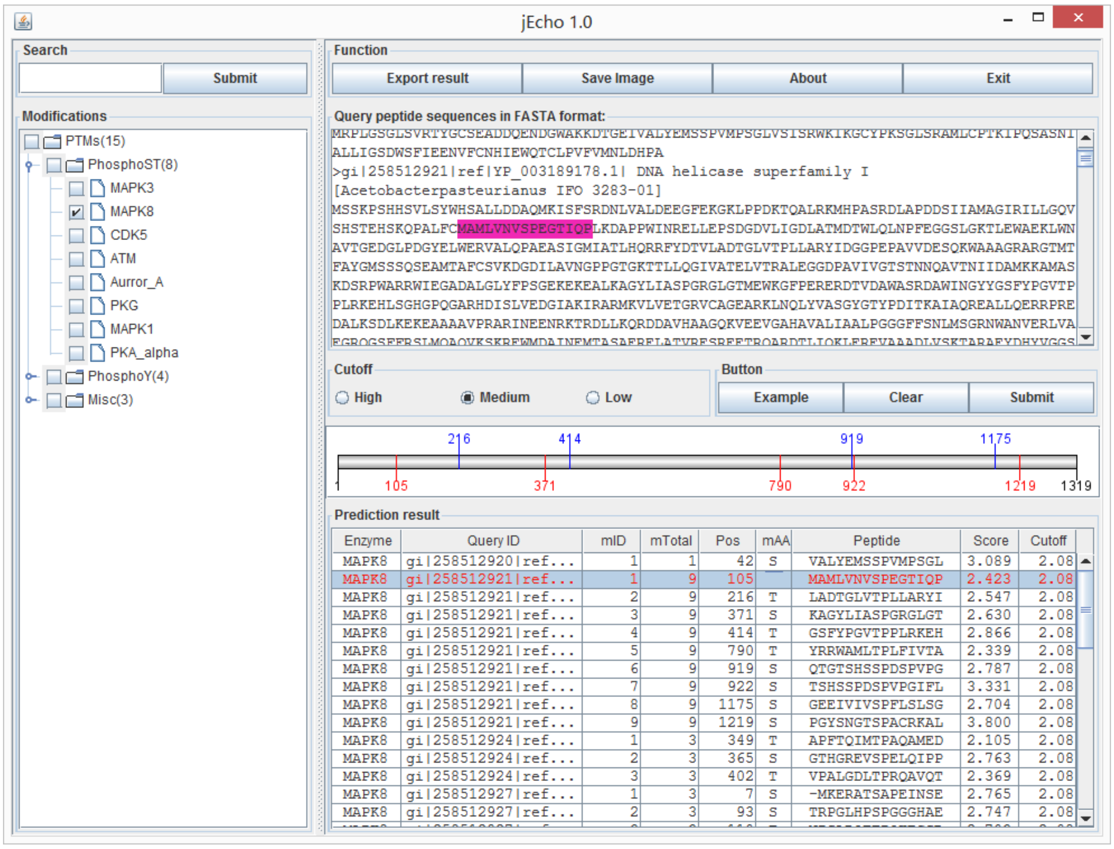
jEcho 1.0
Search
Submit
Modifications
PTMs(15)
PhosphoST(8)
MAPK3
MAPK8
CDK5
ATM
Aurror_A
PKG
MAPK1
PKA_alpha
PhosphoY(4)
Misc(3)
Function
Export result
Save Image
About
Exit
Query peptide sequences in FASTA format:
Cutoff
High
Medium
Low
Button
Example
Clear
Submit
Prediction result
Enzyme
Query ID
mID
mTotal
Pos
mAA
Peptide
Score
Cutoff

(b)

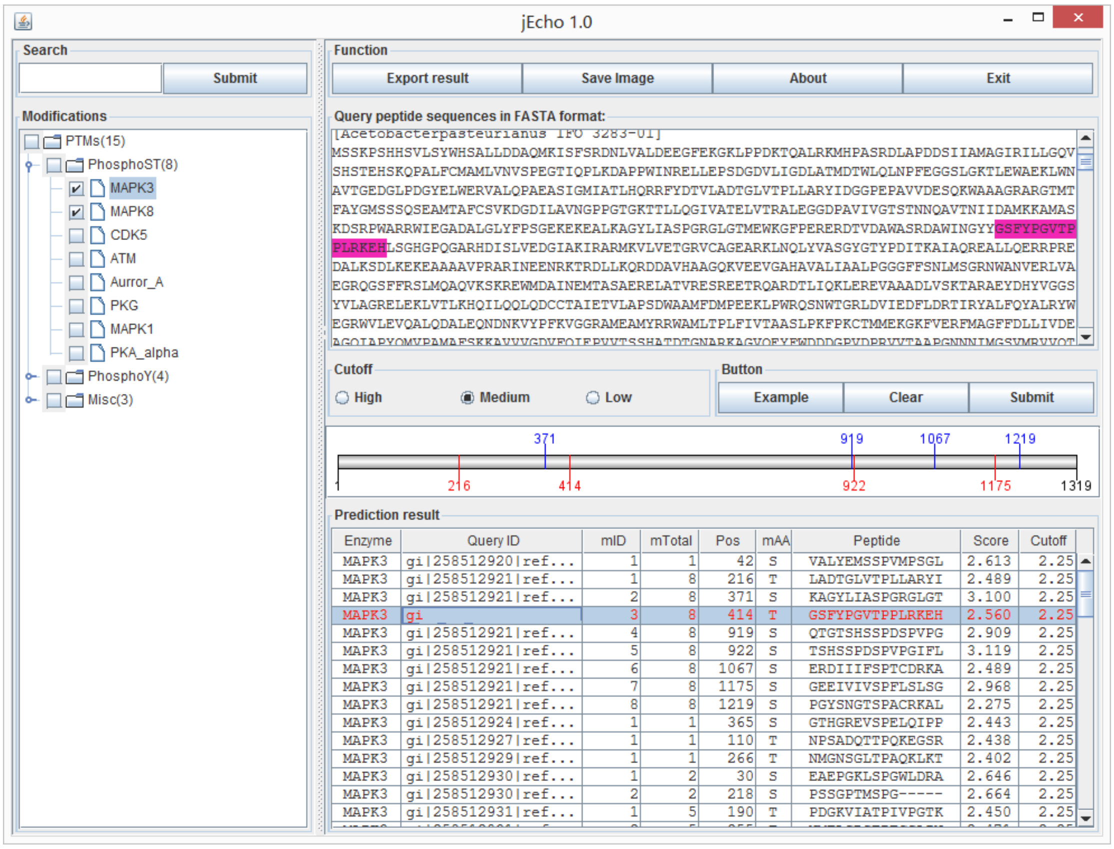

(c)

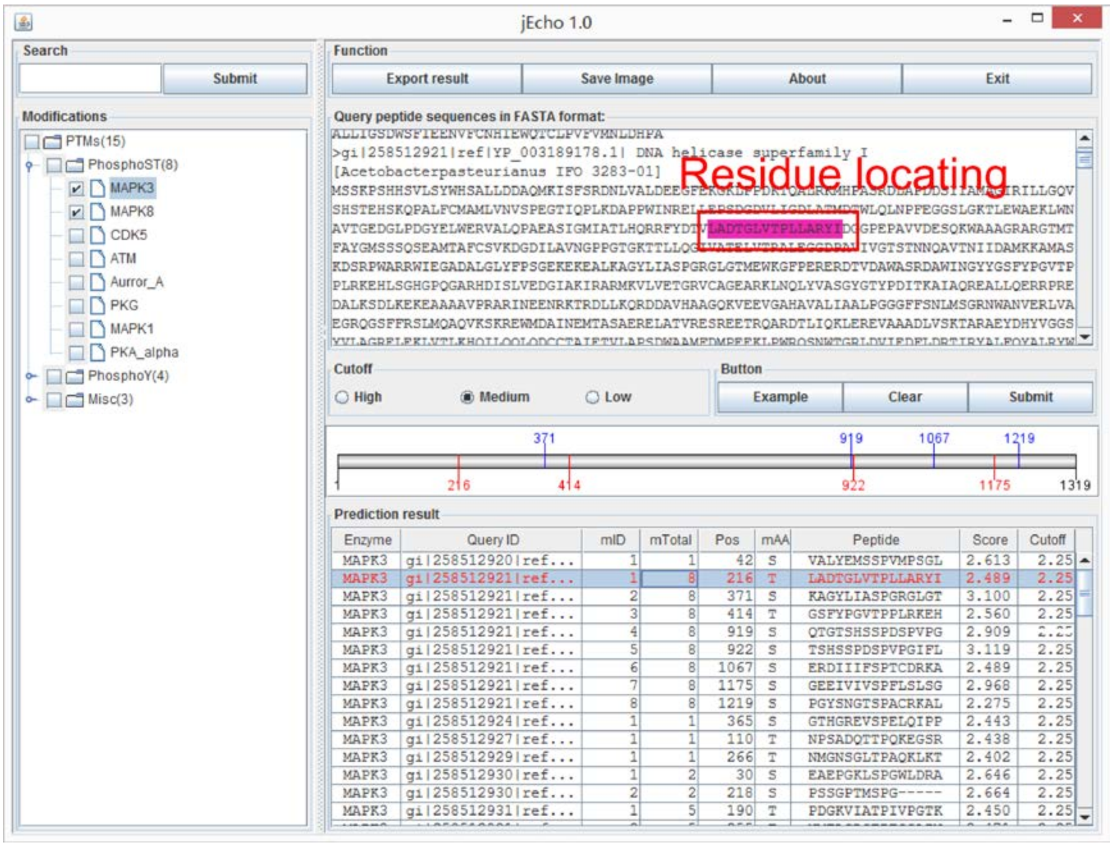
jEcho 1.0
Search
Submit
Function
Export result
Save Image
About
Exit
Modifications
Query peptide sequences in FASTA format:
Residue locating
Cutoff
High
Medium
Low
Button
Example
Clear
Submit
Prediction result
Enzyme
Query ID
mID
mTotal
Pos
mAA
Peptide
Score
Cutoff

(d)

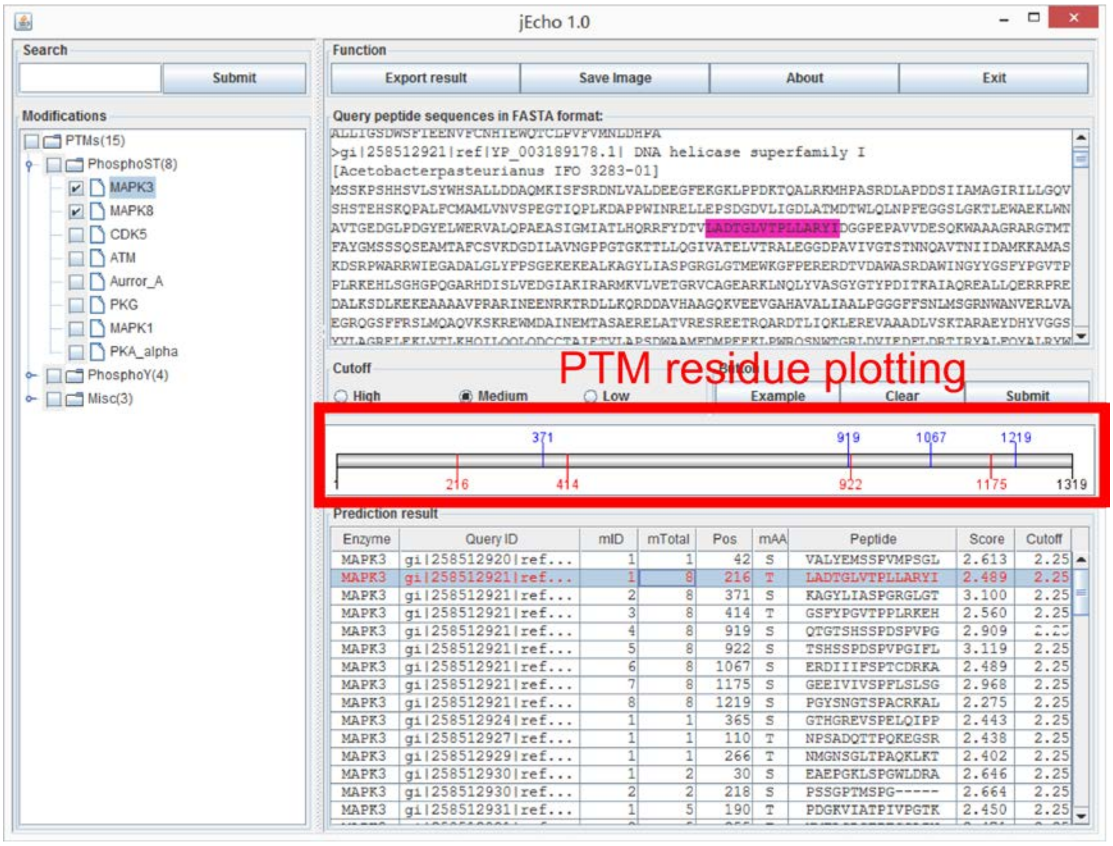
jEcho 1.0
Search
Submit
Modifications
PTMs(15)
PhosphoST(8)
MAPK3
MAPK8
CDK5
ATM
Aurror_A
PKG
MAPK1
PKA_alpha
PhosphoY(4)
Misc(3)
Function
Export result
Save Image
About
Exit
Query peptide sequences in FASTA format:
>gi|258512921|ref|YP_003189178.1| DNA helicase superfamily I [Acetobacterpasteurianus IFO 3283-01]
Cutoff
High
Medium
Low
Example
Clear
Submit
PTM residue plotting
371
919
1067
1219
1
216
414
922
1175
1319
Prediction result
Enzyme | Query ID | mID | mTotal | Pos | mAA | Peptide | Score | Cutoff
MAPK3 | gi|258512920|ref... | 1 | 1 | 42 | S | VALYEMSSPVMPSGL | 2.613 | 2.25
MAPK3 | gi|258512921|ref... | 1 | 8 | 216 | T | LADTGLVTPLLARYI | 2.489 | 2.25
MAPK3 | gi|258512921|ref... | 2 | 8 | 371 | S | KAGYLIASPGRGLGT | 3.100 | 2.25
MAPK3 | gi|258512921|ref... | 3 | 8 | 414 | T | GSFYPGVTPPLRKEH | 2.560 | 2.25
MAPK3 | gi|258512921|ref... | 4 | 8 | 919 | S | QTGTSHSSPDSPVPG | 2.909 | 2.25
MAPK3 | gi|258512921|ref... | 5 | 8 | 922 | S | TSHSSPDSPVPGIFL | 3.119 | 2.25
MAPK3 | gi|258512921|ref... | 6 | 8 | 1067 | S | ERDIIIFSPTCDRKA | 2.489 | 2.25
MAPK3 | gi|258512921|ref... | 7 | 8 | 1175 | S | GEEIVIVSPFLSLSG | 2.968 | 2.25
MAPK3 | gi|258512921|ref... | 8 | 8 | 1219 | S | PGYSNGTSPACRKAL | 2.275 | 2.25
MAPK3 | gi|258512924|ref... | 1 | 1 | 365 | S | GTHGREVSPELQIPP | 2.443 | 2.25
MAPK3 | gi|258512927|ref... | 1 | 1 | 110 | T | NPSADQTTPQKEGSR | 2.438 | 2.25
MAPK3 | gi|258512929|ref... | 1 | 1 | 266 | T | NMGNSGLTPAQKLKT | 2.402 | 2.25
MAPK3 | gi|258512930|ref... | 1 | 2 | 30 | S | EAEPGKLSPGWLDRA | 2.646 | 2.25
MAPK3 | gi|258512930|ref... | 2 | 2 | 218 | S | PSSGPTMSPG----- | 2.664 | 2.25
MAPK3 | gi|258512931|ref... | 1 | 5 | 190 | T | PDGKVIATPIVPGTK | 2.450 | 2.25

(e)

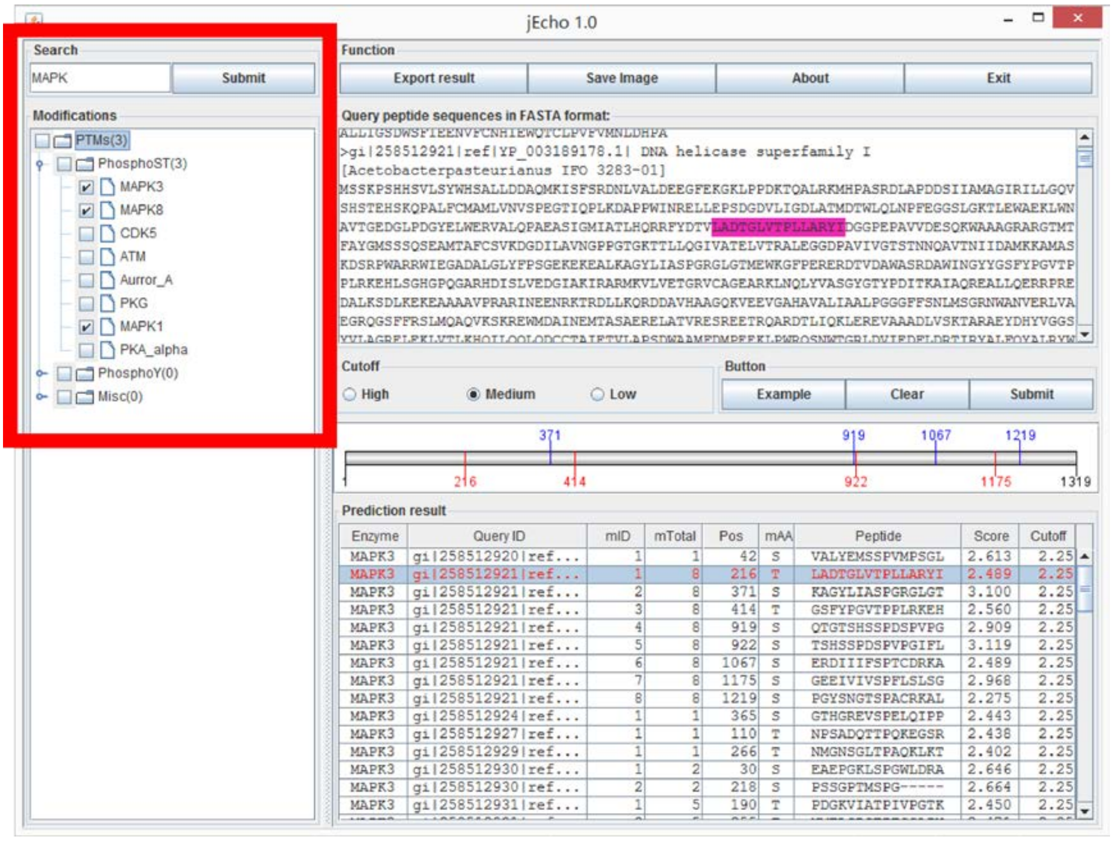
jEcho 1.0
Search
MAPK
Submit
Modifications
PTMs(3)
PhosphoST(3)
MAPK3
MAPK8
CDK5
ATM
Aurror_A
PKG
MAPK1
PKA_alpha
PhosphoY(0)
Misc(0)
Function
Export result
Save Image
About
Exit
Query peptide sequences in FASTA format:
Cutoff
High
Medium
Low
Button
Example
Clear
Submit
Prediction result
Enzyme
Query ID
mID
mTotal
Pos
mAA
Peptide
Score
Cutoff

(f)

jEcho's user guide

In order to facilitate the user-friendly usage of these models, we implement the Echo algorithm and its nine classification models as the computer program jEcho version 1.0 (Figure 1). The programming environment is Eclipse version 4.2.1, with the Java Running Environment version 1.7.0_21. We do not use any latest technology. So jEcho version 1.0 should run under any Java versions across different operating systems.

By following the suggested functionalities of a PTM prediction server/program [1], we design the user interface of jEcho as the style of all functions in one window, as shown in Figure 1 and Supplementary Figure S1 (a). The user may get a quick demo by clicking the "Example" button, which loads two protein sequences from *Acetobacter pasteurianus* IFO 3283-01 into the query sequence box in the top right corner of the window. To make the demo simpler, the selection of kinase MAPK8 will also be automatically chosen, as shown in Supplementary Figure S1 (b). After clicking the "Submit" button, 9 predicted phosphoserine/threonine residues are detected in the two protein sequences, and listed in the result table box in the bottom right corner.

A number of productivity tools are provided to facilitate biologist's exploration of the predicted PTM residues. The function of "Sort" is provided to help the investigation of residues modified by different PTM types, as shown in Supplementary Figure S1 (c). The user may investigate all the PTM residues in a given protein, by sorting the prediction result lines with the column "Query ID". An investigation of all the residues modified by a given PTM may be conducted by clicking the column "Enzyme". The function of "residue locating" is provided to help the fast locating of the candidate PTM residue and its flanking peptide, by clicking the given line of predicted PTM residue, as shown in Figure S1 (d). Sometimes there may be dozens of predicted PTM residues in multiple user-input sequences, and this function will help the user easily find the predicted residue and the flanking region. The function of "PTM residue plotting" is provided to demonstrate the distribution of predicted PTM residues in the current protein sequence, as shown in Figure S1 (e). The function of "PTM type searching" is provided to help the user quickly find the PTM types of interest, as shown in Supplementary Figure S1 (f).

After the final choices of PTM types and target protein sequences, all the results in jEcho may be exported for further analysis or publication purpose. The candidate PTM residues in the bottom right box may be exported as a TEXT file or a PDF file by clicking the "Export result" button. The results in the TEXT format may be used for large-scale association analysis. The distribution plot of candidate PTM residues in a protein may be saved as a vector image (SVG format) or a pixel image (JPG format). The SVG image may be processed and saved as a pixel image with publication quality resolution (*e.g.* 300 dpi) by the computer programs Inkscape (free GPL2 license) or Adobe Illustrator (commercial license). The JPG format may be processed by the programs ImageMagik (free Apache 2.0 license) or Adobe Photoshop (commercial license).

References

1. Zhou F, Xue Y, Yao X, Xu Y: **A general user interface for prediction servers of proteins' post-translational modification sites**. *Nature protocols* 2006, **1**(3):1318-1321.
